# Book Reviews

**Published:** 1899-09

**Authors:** 


### Book Reviews.

---

THE PRINCIPLES OF BACTERIOLOGY. A practical manual for students and physicians. By A. C. Abbott, M. D., Professor of Hygiene and Director of the Laboratory of Hygiene, University of Pennsylvania, Philadelphia. New (fifth) edition, enlarged and thoroughly revised. Duodecimo, 585 pages, 109 illustrations, of which twenty-six are colored. Philadelphia and New York: Lea Brothers & Company.

The popularity of Abbott's treatise is accounted for largely from the fact that it is so practical in character. When five editions are demanded of a work on bacteriology within eight years, is is pretty complete evidence that it has taken hold of the student and teacher. Abbott, in our view, has done more than any other author to popularise the study of bacteriology by the general practitioner of medicine.

A certain amount of bacteriological knowledge is essential to the intelligent practice of medicine in everyday life. Neither specialist nor general physician can get along smoothly with his work without this important aid to diagnosis, and we had almost said that he cannot proceed to an intelligent understanding of the subject without Abbott's book. At all events, we feel justified in affirming that this treatise will simplify the practical pursuit of the study of bacteriology.

The present edition has been revised to date, and many chapters, particularly those upon technique, disinfection, specific infections, immunity, and the like, have been enlarged. Equally thorough has been the revision and improvement of the illustrations, both black and colored.

Confidence in a still greater demand for this new edition is shown in the fact that notwithstanding an increase of some 50 pages, and the addition of illustrations in black and colors, the price of the book has not been advanced.

---

TRANSACTIONS OF THE SOUTHERN SURGICAL AND GYNECOLOGICAL ASSOCIATION. Eleventh annual session, held at Memphis, Tenn., December 6, 7 and 8, 1898. William E. B. Davis, M. D., Secretary.

The value of preserving society proceedings in permanent form is demonstrated again and again by the admirable report that comes to us year by year of the transactions of this staunch and progressive association. We are constrained to make this remark from the fact that many medical societies have established journals as their organs and put forth their transactions mixed with other matter in periodical form. It seems to us there are several objections to journalising the proceedings of special societies, the chiefest of which is that such a plan deprives the searcher of the literature of any particular subject, or group of subjects, of the opportunity of finding readily that information for which he is on the lookout. The completeness of a single volume wherein a year's work of distinguished specialists may be found carefully recorded, well printed and properly indexed, is such a great satisfaction that the experienced writer would be slow to exchange it for a medical journal appearing at various periods throughout the year.

For instance, in the present volume the article on Thoracic resection for tumors growing from the bony wall of the chest, by Professor F. W. Parham, of New Orleans, occupies about 150 pages. It is a most discursive, elaborate and exhaustive monograph and its value would be very materially detracted from were it split up into parts and printed in a medical journal as a serial. If it be argued that society transactions, as a rule, are slow in appearing, a circumstance always due to the tardiness of the members themselves, in furnishing copy or revising proof, it may be answered that very complete abstracts of proceedings, embracing synopses of papers and discussions, appear promptly in some magazine devoted to the specialty which concerns the society interested; whereas, later, the volume is printed giving a complete setting forth of everything said and done at the meeting from the merest interlocutory motion to the completest and most scientific of papers. The society thus has the benefit of a double publication of its work—one, immediate, in quite full abstract, and the other remote and absolutely complete.

The present volume is one of the best this distinguished association ever published, and this in spite of the fact that its last meeting was held in the midst of one of the most remarkable storms that the country has experienced in many years,—one that materially embarrassed travel to and from the meeting.

The book closes with a memorial sketch of Dr. Cornelius Kollock, written by Dr. Richard B. Maury, and is accompanied by an excellent half-tone portrait of this genial and accomplished South Carolinian.

A Treatise on Human Physiology. For the Use of Students and Practitioners of Medicine. By Henry C. Chapman, M. D., Professor of Institutes of Medicine and Medical Jurisprudence in the Jefferson Medical College of Philadelphia. New (second) edition, thoroughly revised. In one handsome 8vo. volume of 921 pages, with 595 engravings. Philadelphia and New York: Lea Brothers & Co. 1899.

Since Dalton, fresh from the teachings of M. Claude Bernard, at Paris, began to instruct his pupils through experimental research, the study of physiology has shot forward with wonderful speed. Now, however, it is not sufficient to present a treatise to student or practitioner of medicine based alone upon laboratory experiments. While the importance of those have not diminished, but are rather increasing with the advance of time, there must be added to this method of investigation, those developed upon comparative and pathological anatomy, clinical medicine, physics and chemistry. Such a work Chapman sometime ago presented to the medical profession and its popularity is well attested by a demand for a second edition so soon after the first was put forth. In this revision the author has adhered to his fundamental plan, but has recast the details. Physiological chemistry has come forward with claims of vast importance that must be recognised, and the functions of the nervous system have become better and better understood with each advancing year, hence important changes and additions have been made, and that which has diminished or ceased in importance has been eliminated. The book, therefore, remains about the same size as at first, and is even more useful as a text-book, while the price has been lowered, bringing it within the reach of all who need it.

In the department wherein he considers the physiology of the nervous system Chapman is particularly strong, and no student or practitioner will be disappointed in following his book in this important branch. He is very interesting, too, in dealing with the special senses and here, as is indeed, all through the book, his English is clear and fluent, and his sentences are terse and full of meaning. The high position already taken by this work will not only be maintained through the excellence of this edition but we feel sure must be greatly enhanced.

THE SERUM DIAGNOSIS OF DISEASE. By RICHARD CABOT, M. D., Physician to Out-patients, Massachusetts General Hospital. Small 8vo, pp. vii.—154. New York: William Wood & Company. 1899.

This book according to the author's statement is a compilation, its aim being to group in convenient form the vast work that has been done upon serum diagnosis since 1896. Impressed with the importance of pursuing further investigation upon the subject as related to fevers rife among the soldiers in our new possessions, the author went to Cuba and Porto Rico last year and took up the investigation for himself. In the United States general hospital, at Ponce, he found several hundred fever cases nearly all of whom were receiving large doses of quinine simply because the diagnosis between typhoid and malaria had not been established. Proceeding with the investigation Cabot examined 177 cases at the hospital in question where, strange to say, he found not a single case of malaria among them. On the other hand he states that 90 per cent. of those examined showed prompt and intense serum reaction for typhoid disease. Upon this evidence, supplemented with that previously obtained in fifteen cases by the pathologist of the hospital, the wholesale administration of quinine was checked and the typhoid cases needing diet were indicated. We mention this merely to emphasise the important part serum not only plays in diagnosis but in indicating the line of treatment to be pursued.

This method of diagnosis is yet in the hands of comparatively few men, and it is to stimulate its more general use among general practitioners of medicine that Cabot has prepared this book. In it he has only recorded facts already known or established, avoiding as far as possible the theoretical. This brings it well within the range of safety as a guide to general practitioners of medicine, to whom we especially commend it. It contains a complete bibliographical list up to the present year and all through the work the references are numerous.

---

CHEMISTRY: General, Medical and Pharmaceutical, including the Chemistry of the U. S. Pharmacopeia. By JOHN ATTFIELD, F. R. S. Sixteenth edition. In one royal 12mo volume of 784 pages, with eighty-eight illustrations. Philadelphia and New York: Lea Brothers & Company. 1899.

This standard work, true to its traditions, again appears with all its essentials brought forward to the immediate present. The frequency with which the several editions of the book have been brought out has enabled the author to keep pace with events and to the claims of this treatise all the freshness of a new work by a new author. At the same time, too, it possesses the additional advantages of being the consensus of years of experience in teaching and writing upon chemistry.

This edition is adapted to both the pharmacopeia of the United States and that of Great Britain. When we consider the great strides made in chemistry within the last few years, the task of keeping such a work well to the fore and at the same time eliminating whatever as been superseded or become obsolete, is not an easy one.

That the author has done all this and more, will be acknowledged by those familiar with the successive editions, as they have appeared. The size of the volume, however, still remains within the limits belonging to a manual.

The special advantage of this work is its adaptability to medical and pharmaceutical needs, making it valuable to both student and practitioner of medicine. Its voluminous index makes its contents readily accessible.

